# Detection of antimicrobial resistance genes in urban air

**DOI:** 10.1002/mbo3.1248

**Published:** 2021-11-16

**Authors:** Ágnes Becsei, Norbert Solymosi, István Csabai, Donát Magyar

**Affiliations:** ^1^ Department of Physics of Complex Systems Eötvös Loránd University Budapest Hungary; ^2^ Centre for Bioinformatics University of Veterinary Medicine Budapest Hungary; ^3^ National Public Health Center Budapest Hungary

**Keywords:** air metagenomics, ARG, bacteriome, Harvard impactor, PM

## Abstract

To understand antibiotic resistance in pathogenic bacteria, we need to monitor environmental microbes as reservoirs of antimicrobial resistance genes (ARGs). These bacteria are present in the air and can be investigated with the whole metagenome shotgun sequencing approach. This study aimed to investigate the feasibility of a method for metagenomic analysis of microbial composition and ARGs in the outdoor air. Air samples were collected with a Harvard impactor in the PM_10_ range at 50 m from a hospital in Budapest. From the DNA yielded from samples of PM_10_ fraction single‐end reads were generated with an Ion Torrent sequencer. During the metagenomic analysis, reads were classified taxonomically. The core bacteriome was defined. Reads were assembled to contigs and the ARG content was analyzed. The dominant genera in the core bacteriome were *Bacillus*, *Acinetobacter*, *Leclercia* and *Paenibacillus*. Among the identified ARGs best hits were *vanRA*, *Bla1*, *mphL*, *Escherichia coli* EF‐Tu mutants conferring resistance to pulvomycin; *BcI*, *FosB*, and *mphM*. Despite the low DNA content of the samples of PM_10_ fraction, the number of detected airborne ARGs was surprisingly high.

## Graphical abstract

A standardized method for the metagenomic analysis of airborne samples would help to estimate the public health risks of airborne antimicrobial resistance genes (ARGs). We conducted a metagenomic analysis to reveal the core bacteriome and the ARG profile of three urban air samples collected with a Harvard impactor. The core bacteriome was dominated by *Bacillus*, *Acinetobacter*, *Leclercia* and *Paenibacillus* species. We detected several antimicrobial resistance genes. All of them were associated with non‐pathogenic bacteria.

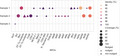

## 1 INTRODUCTION

Antimicrobial resistance (AMR) is one of the top global public health threats worldwide. Widespread misuse and overuse of antimicrobial drugs are accelerating the evolution and selection of naturally occurring antimicrobial resistance genes (ARGs) in bacteria (Vikesland et al., 2019). In hospitals drug‐resistant pathogenic bacteria are widespread, however, environmental microbes in the soil, water, air, or animal microbiome act as ample reservoirs of ARGs accumulated over time. The current understanding of AMR is derived from culture‐based and phenotypic methods. These methods only aim at a few mostly pathogenic bacteria and do not detect the majority of ARGs in environmental microbes (Be et al., 2014). Culture‐independent approaches, like 16S rRNA gene sequencing, provide taxonomic identification usually only on the genus level. Quantitative real‐time polymerase chain reaction (PCR) analysis of ARGs only targets a limited number of genes (Echeverria‐Palencia et al., 2017; Fluit et al., 2001). Whole‐genome shotgun sequencing provides suitable data for metagenomic analysis. There are many metagenomic studies on the investigation of bacteria and genes in samples like water or soil, but there are only a few studies analyzing air samples (Aalismail et al., 2019; Be et al., 2014, 2017; King et al., 2016). Airborne microbes carrying ARGs are attached to solid particles and liquid droplets constituting a mixture called particulate matter (PM). PM circulates in the air for a long time and travels long distances. These particles vary in size, therefore the health risk of PM_2.5_ and PM_10_ is significant because these sizes correspond to human inhalable particles (<10 µm; Liu et al., 2018). Usually collecting a proper amount of genetic material for air metagenomics studies is challenging. However, there is a wide range of sampling and sequencing methods with various efficacy available for air metagenomics studies, but without standards and best practices interpretation across these studies is difficult. In this study, we aim to investigate the sensitivity of a method to detect airborne ARGs and to examine the airborne microbial community in an outdoor urban environment.

## 2 MATERIALS AND METHODS

Three air samples were collected with a Harvard impactor (Marple et al., 1987) in the PM_10_ size range, with a flow rate of 10 L/min, a total of 44.4 m^3^ of air onto 37 mm fiberglass filters (Whatman GF/A; GE Healthcare Life Science) for 3 × 5 days in September 2019. The samples were collected in the open air, 50 m from the entrance of a hospital in Budapest, Hungary, which specializes in the treatment of infectious diseases. Total DNA was then extracted from the pellets using the ZR Fecal DNA Kit (Zymo Research). Single‐end reads were generated by an Ion Torrent Sequencer. Quality‐based filtering was performed by Trimmomatic (Bolger et al., 2014) with 20 as a quality threshold for bases and with retaining reads with a minimum length of 50 bp. Replicates were removed by vsearch 2.14.2 (Rognes et al., 2016). Filtered reads were taxonomically classified by Kraken 2 (Wood et al., 2019), (*k* = 35), using the National Center for Biotechnology Information non‐redundant nucleotide database (Pruitt et al., 2005). Bacterial reads were assembled by metaSPADES 3.14.1 (Nurk et al., 2017) with an automatically estimated maximum k‐mer size of 127. Protein sequences of open reading frames (ORFs) were predicted by Prodigal setting “meta” mode for metagenome. The ARG content of the ORFs was identified by the Resistance Gene Identifier v5.10 (Alcock et al., 2019) using “The Comprehensive Antibiotic Resistance Database” (CARD) v.3.0.6 (Alcock et al., 2019). "Perfect" hits are protein sequences with 100% match to CARD reference sequences, while the ‘strict’ category is more flexible allowing some variation from the CARD reference sequence. "Loose" hits fall out of the detection model cut‐offs. All "Loose" hits with identity ≥95% were nudged to the ‘strict’ category. All ARGs presented here are classified as "strict" hits. Contigs associated with ARGs, with "strict" or "perfect" cut‐offs, were taxonomically classified using Kraken 2 the same way as described above.

## 3 RESULTS AND DISCUSSION

### 3.1 Bacteriome

In this study, sampling procedures for all three samples were alike, except that the samples were collected one after the other, indicating a probable diversity of PM content and composition. The average PM_10_ concentration in sample 1 was 24.4 µg/m^3^, 25.36 µg/m^3^ in sample 2, and 42.9 µg/m^3^ in sample 3. A study focusing on bacteria in aerosols showed strong fluctuations that correlated significantly with changes in seasonal temperatures (Ravva et al., 2012). In another study, relative humidity and PM_10_ were the key factors that significantly affected the airborne bacterial concentration and community structure (Li et al., 2018). Therefore, it is reasonable to assume that these were the factors that caused large fluctuations in abundances of airborne particulate matter and thereby cause fluctuations in the concentration of bacteria in our samples.

**FIGURE 1:**
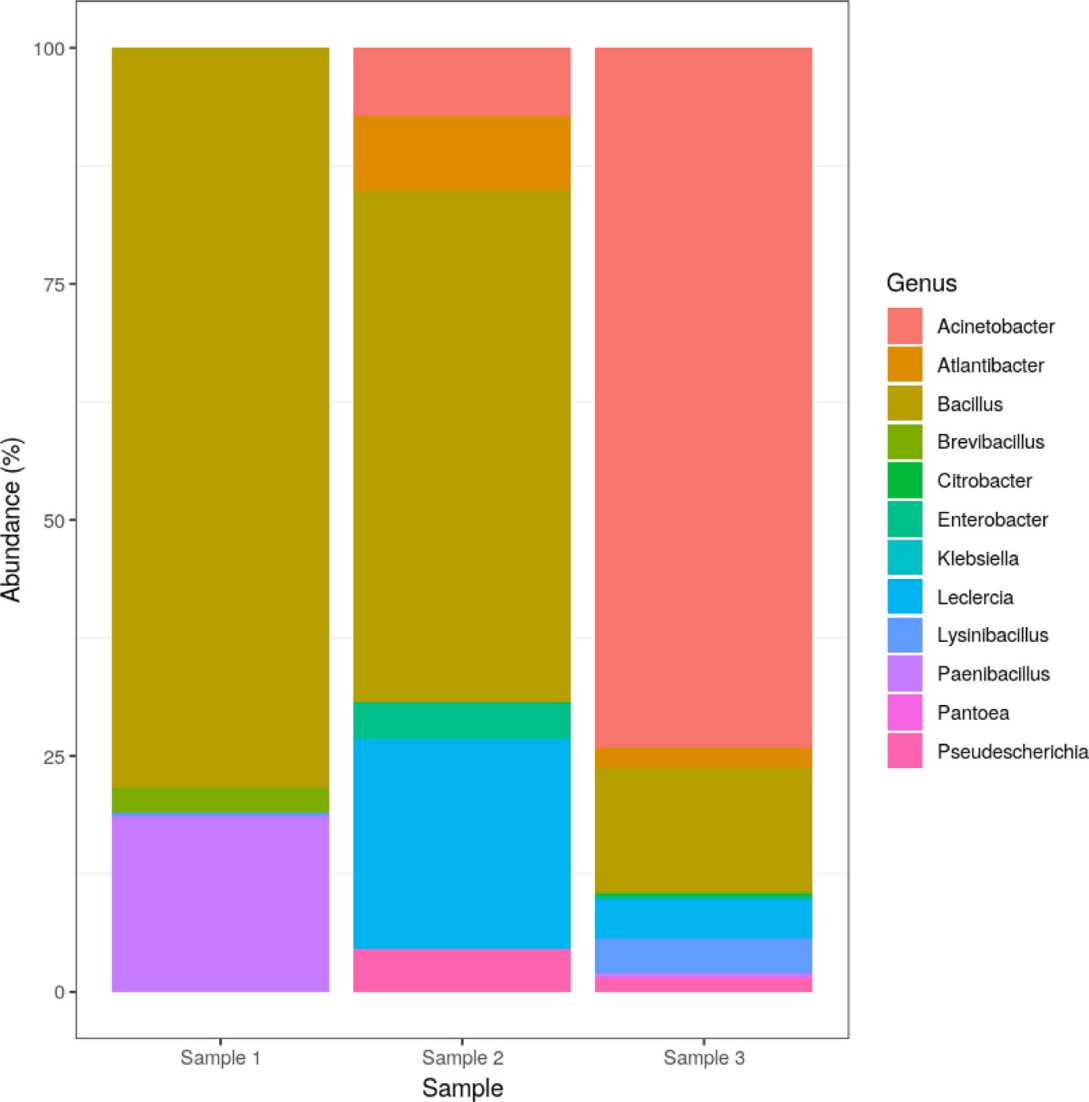
Relative abundances of the most common bacterial genera. Core bacteriome was defined as the relative abundance of agglomerated counts at the genus level above 0.0005 and with prevalence above 0.6. Core bacteriome is dominated by the genera *Bacillus*, *Acinetobacter*, *Leclercia* and *Paenibacillus*.

Sequencing resulted in 855,654 single‐end reads in sample 1, 2,290,392 reads in sample 2, and 527,221 reads in sample 3. By prefiltering steps, 19.57% of sample 1, 21.92% of sample 2, and 42.95% of sample 3 were discarded. Taxonomic classification was successful with 95.52% of the reads in sample 1, 94.11% in sample 2, and 84.79% in sample 3. Taxon classification of reads revealed that most classified reads are aligned to bacterial genomes. Dominant phyla were Firmicutes and Proteobacteria, which are rather common in air samples (Aalismail et al., 2019; Be et al., 2014; Yooseph et al., 2013). The most abundant genera are the *Bacillus*, *Acinetobacter*, *Leclercia* and *Paenibacillus* (Figure 1). Members of the genus *Bacillus* are among the most abundant in sample 1 and sample 2 with species of the *Bacillus cereus* group (Figure 1). They were also the most abundant inhabitants of urban air in another study (Be et al., 2014).

In sample 3, the most abundant genus was *Acinetobacter* (Figure 1). It is a highly diverse group of mostly non‐pathogenic environmental microbes isolated from samples like soil and wastewater. They often carry several ARGs including carbapenemases and extended‐spectrum beta‐lactamases (Wong et al., 2017). Other abundant genera in core bacteriome are *Atlantibacter*, *Citrobacter*, *Enterobacter*, *Klebsiella*, and *Pseudoescherichia* (Figure 1) belonging to the diverse family *Enterobacteriaceae* (Morales‐López et al., 2019).

### 3.2 Antimicrobial resistance genes

The total number of assembled contigs in sample 1 was 7613, 1137 in sample 2, and 235 in sample 3. In these contigs, 12 ARGs were identified in sample 1, 13 ARGs in sample 2, and 1 ARG in sample 3. The median lengths of assembled contigs are 660 (interquartile range, IQR: 510) in sample 1, 698 (IQR: 644.5) in sample 2, and 505 (IQR: 104.5) in sample 3. The mean coverage of the listed ARGs in sample 1 is 43.27% with a range of 3.91%–102.27% and mean identity is 97.74% with a range of 87.71%–100%. In sample 2, the coverage of ARG hits ranged between 7.46% and 108.7% with a mean value of 49.65%. The range of identity values is between 90.91% and 100% with a mean value of 96.57%. Sample 3 resulted in only one ARG with 100% identity and 3.97% coverage.

**FIGURE 2:**
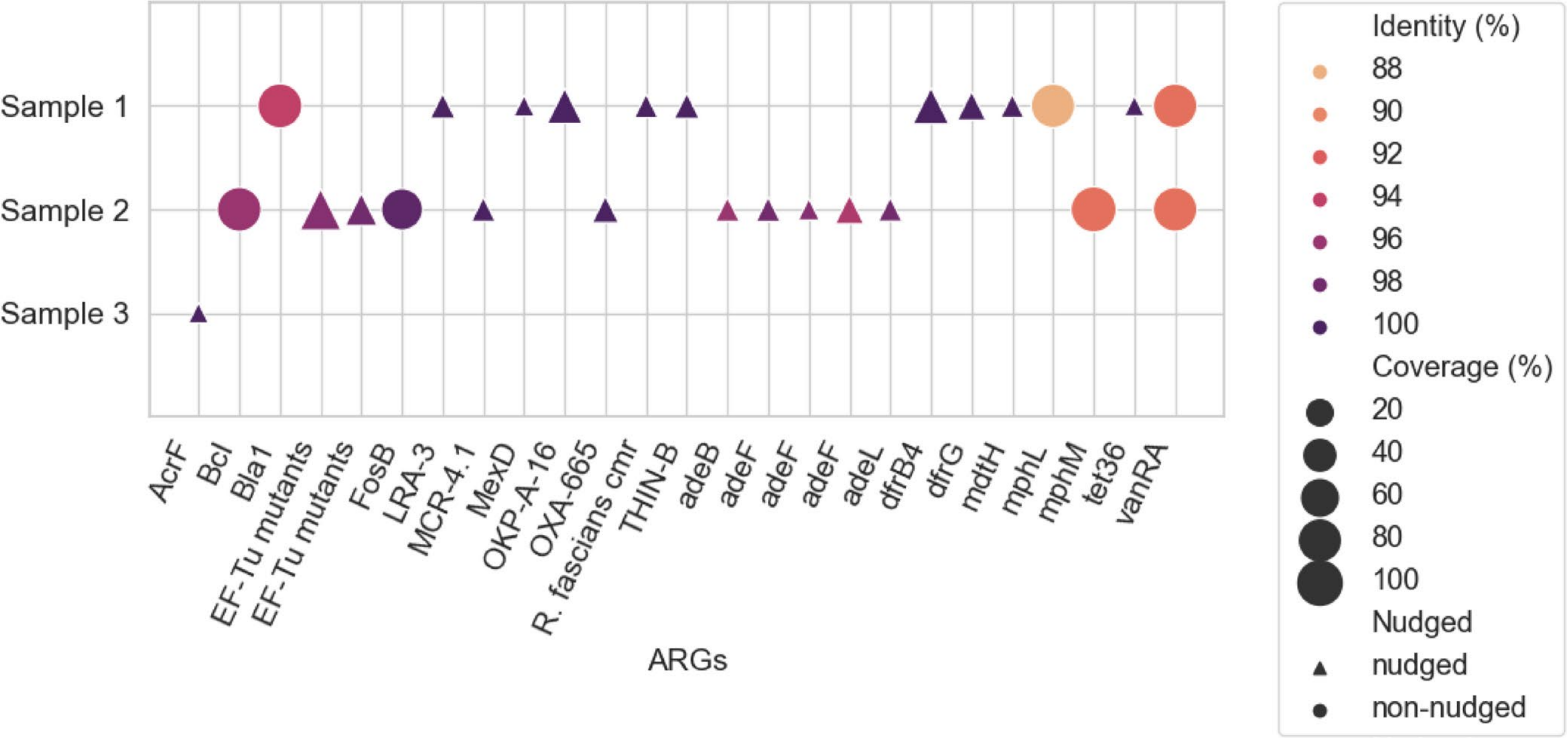
Identity and coverage of detected ARGs. All ARGs presented here are classified as “strict” hits. Different symbols stand for nudged or non‐nudged hits from the ”loose” to ”strict” category. The color of the symbols corresponds to the percentage of identity of the top ARG hit. The size of the points corresponds to the ratio of length between contig and the CARD reference sequence. ”EF‐Tu mutants” refers to *Escherichia coli* EF‐Tu mutants conferring resistance to pulvomycin and ”R. fascians cmr” to *Rhodococcus fascians cmr*. ARGs, antimicrobial resistance genes

Among the best hits in our samples, *vanRA,* also known as *vanR* (Figure 2), together with *vanS*, is part of the regulatory system of the *vanA* resistance gene cluster responsible for peptidoglycan target alteration of the glycopeptide antibiotic, vancomycin (Courvalin, 2006).

*Bla1* and *BcI* are beta‐lactamase genes detected with coverage and identity values near 90% (Figure 2). *Bla1* codes a penicillinase, first recognized in *Bacillus anthracis* (Materon et al., 2003). Note, *BcI* codes a zinc metallo‐beta‐lactamase associated with *B*. *cereus* that hydrolyses many penicillins including carbapenems which generally escape from serine beta‐lactamases (Carfi et al., 1995). With high coverage and identity values, *mphL* and *mphM* (Figure 2) are expressed as macrolide phosphotransferases which are also highly prevalent in members of the *B. cereus* group (Wang et al., 2015). Another ARG, with near 90% coverage and identity is *Escherichia coli* EF‐Tu mutants conferring resistance to pulvomycin (Figure 2). Pulvomycin inhibits protein synthesis by acting on elongation factor Tu (EF‐Tu). In *E. coli* *,*EF‐Tu is very sensitive to pulvomycin, but membrane impermeability of Gram‐negative bacteria prohibits several antibiotics, including pulvomycin, to enter the cell. Maybe as a second line of defense, in case of increased permeability, non‐sensitive EF‐Tu mutants are more resistant to pulvomycin than the wild‐type (Zeef et al., 1994). In another resistance mechanism, the protein encoded by FosB (Figure 2) is a Mn^2+^‐dependent enzyme that modifies fosfomycin to a compound with no bactericidal properties (Thompson et al., 2013). Other hits shown in the figure with lower coverage and identity values are probably variants of ARG reference sequences in CARD.

**FIGURE 3:**
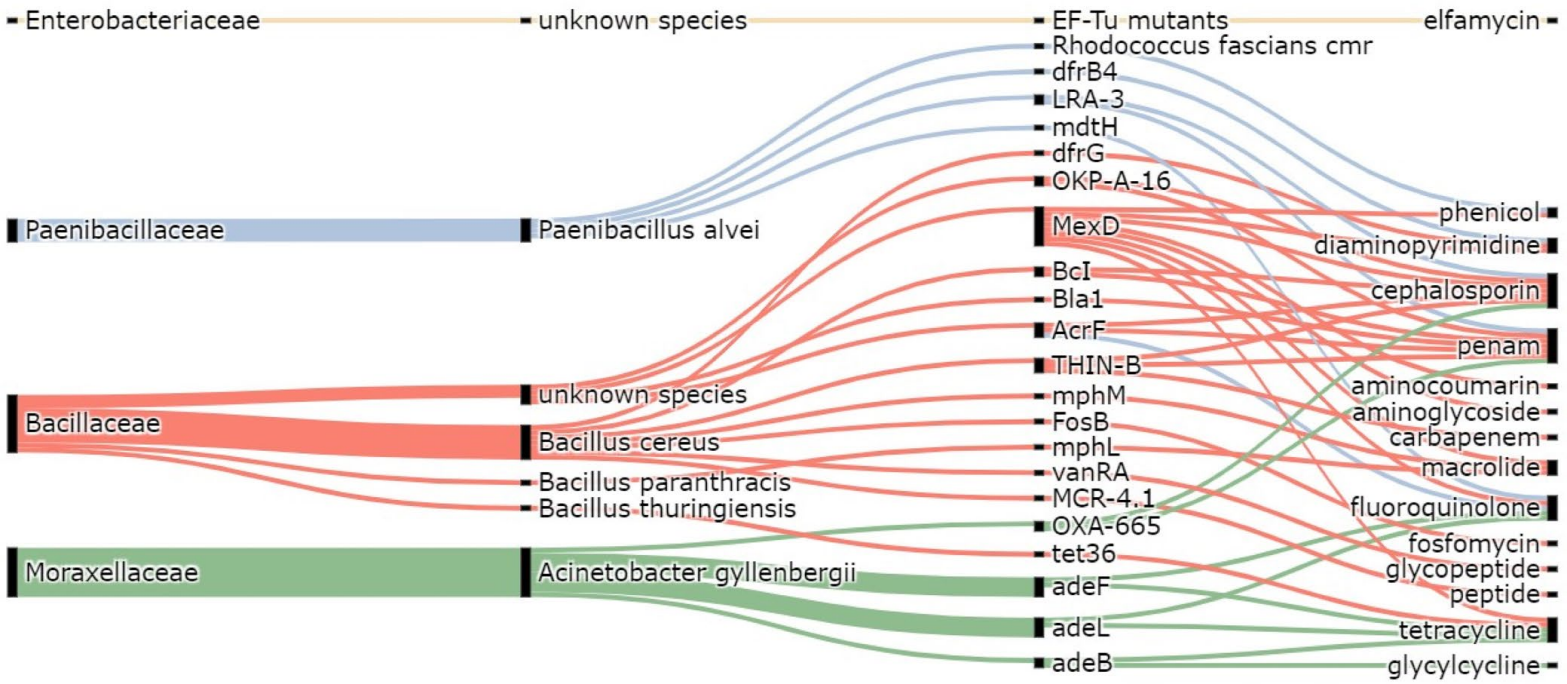
Identified ARGs, their most probable taxon of origin, and the group of antibiotics they protect against. One gene probably originated from a member of the family *Enterobacteriaceae*, four genes from *Paenibacillus alvei*, three from unknown members of the family *Bacillaceae*, six from *Bacillus cereus*, from *B*. *paraanthracis* and *B*. *thuringiensis*, one each and four from *Acinetobacter gyllenbergii*. Most of the detected genes protect beta‐lactams (penams, carbapenems, cephalosporins).

In sample 1, 33% of the detected ARGs probably originated from the genus *Paenibacillus* and 58.3% from the genus *Bacillus*. In sample 2, revealed ARGs probably originated from *Acinetobacter* (46%), *Bacillus* (38%) genera, and the family *Enterobacteriaceae* (15%) (Figure 3).

Non‐culture‐based methods are dominated by PCR techniques (Hu et al., 2018; Li et al., 2018; Xie et al., 2018) and there are only a few studies on airborne ARGs revealed by metagenomic analysis (Fondi et al., 2016; Pal et al., 2016). One of the studies on the investigation of 39 ARG subtypes by PCR in the air of 19 global cities revealed that the most abundant ARGs provide resistance against beta‐lactams, quinolones, macrolides, aminoglycosides, and vancomycin (Li et al., 2018). Similarly, in our result, most ARGs are involved in protection against beta‐lactams, tetracyclines, quinolones, macrolides, and diaminopyrimidines (Figure 3). Although there were several hospitals with intensive use of antibiotics in the vicinity of the sampling site, the closest one specialized in for treatment of infectious diseases. No contigs containing ARGs associated with pathogenic species or strains were identified in any of the air samples. Environmental bacteria as potential reservoirs of ARGs are worth further investigation as developing a standardized method for the metagenomic analysis of airborne samples would help to estimate the public health risks of airborne ARGs.

## CONFLICT OF INTEREST

None declared.

## AUTHOR CONTRIBUTIONS

**Ágnes Becsei:** Formal analysis (equal); software (equal); visualization (equal); writing – original draft (equal). **Norbert Solymosi:** Conceptualization (equal); formal analysis (equal); methodology (equal); software (equal); writing – review & editing (equal). **István Csabai:** Conceptualization (equal); funding acquisition (equal); methodology (equal); supervision (equal); writing – review & editing (equal). **Donát Magyar:** Conceptualization (equal); investigation (equal); methodology (equal); supervision (equal); writing – review & editing (equal).

## ETHICS STATEMENT

None required.

## Data Availability

The datasets generated and analyzed during the current study are available in the NCBI repository at https://www.ncbi.nlm.nih.gov/bioproject/PRJNA747808.
